# Examining Associations Between Individual Exercise, Parent–Child Exercise, and Children’s Mental Health: A Structural Equation Modeling Approach

**DOI:** 10.3390/bs15101353

**Published:** 2025-10-03

**Authors:** Shengsheng Li, Xuanxuan Zhou, Shan Lu, Zhen Xie, Yijuan Lu, Sinuo Wang

**Affiliations:** School of Physical Education, Hangzhou Normal University, Hangzhou 310000, China; 2022210601011@stu.hznu.edu.cn (S.L.); 2023210118140@stu.hznu.edu.cn (X.Z.); 2022212501013@stu.hznu.edu.cn (S.L.); 2023212501116@stu.hznu.edu.cn (Z.X.); 2023210118078@stu.hznu.edu.cn (S.W.)

**Keywords:** parent–child exercise, individual exercise, anxiety, depression, children

## Abstract

**Objective**: This study explores the associations between parent–child exercise and children’s mental health from the perspective of family physical education. **Methods**: In total, 527 valid questionnaires were collected from students in grades four to six of three primary schools in Yuhang District, Hangzhou City, including a survey of the status of children’s exercise and family sports and the SCL-90 symptom self-measurement scale. Based on an analysis of practical challenges in family sports engagement and children’s mental health status, the data were analyzed and modeled using structural equation modeling to obtain a model of children’s mental health promotion, with individual children’s exercise as the primary factor and parent–child exercise as the mediator. **Results:** Both individual children’s exercise and parent–child exercise were significant predictors of children’s mental health promotion. Parent–child activities show a more significant negative correlation with symptoms of anxiety and depression than individual exercise alone. They also partially mediated the relationship between individual exercise and depression/anxiety symptoms. The indirect effects had confidence intervals of [−0.008, −0.001] for depression and [−0.007, −0.001] for anxiety. The direct effects of individual exercise on mental health (depression: β = −0.115; anxiety: β = −0.127) were stronger than the indirect effects and significantly positively correlated with parent–child exercise (β = 0.444, *p* < 0.05), suggesting that individual exercise may encourage more parent–child exercise. **Conclusions:** We propose a relational pathways model incorporating parent–child exercise as a mediating variable and individual exercise as the primary activity. This model is more closely aligned with real-life conditions and practical feasibility than approaches lacking such a family-based component.

## 1. Introduction

In recent years, the physical health status of Chinese children and adolescents has gradually improved. The *Eighth National Survey on Students’ Physical Fitness and Health* shows that morphological development indicators such as the height, weight, and chest circumference of students aged 10–12 have continued to optimize, and physical fitness indicators such as flexibility, strength, speed, and endurance have also shown a positive development trend (China Central Television Website, 2021). However, in sharp contrast, mental health problems among children and adolescents have become increasingly prominent, characterized by younger age of onset, increasing severity, and the complex issue of internet addiction (Lin, 2024), which has become a major challenge in China’s public health field. During the COVID-19 pandemic in particular, intervention measures such as home quarantine and delayed school reopening disrupted the regular rhythms of life and study, significantly exacerbating the occurrence of negative emotions and impulsive behaviors among children (Gu et al., 2023).

Since the 18th National Congress of the Communist Party of China, the Party Central Committee has attached great importance to the mental health and growth of students. The report of the 20th National Congress of the Communist Party of China further proposed to “attach importance to mental health and mental hygiene” (Ministry of Education of the People’s Republic of China, 2023). At present, adolescent mental health issues are facing “new” situations and “new” characteristics, demanding “new” explorations. Existing research data show that 14.8% of children and adolescents may have depressive symptoms to varying degrees, among which 4.0% belong to the “high-risk group for severe depression” and 10.8% belong to the “high-risk group for mild depression”(Fu, 2023). Therefore, exploring scientific and effective intervention paths for children’s mental health has become a key focus in academic and practical circles.

As a non-pharmacological intervention, physical exercise has been proven by numerous empirical studies to have a positive promoting effect on mental health. Moreover, this positive benefit does not follow a single direct “behavior–emotion” mode. Studies have shown that this benefit can be transmitted through intermediate variables such as family interactions (Yang & He, 2024), and this transmission process can be systematically interpreted based on Bandura’s social cognitive theory. This theory emphasizes the triadic reciprocal interaction among individual cognition, behavioral practice, and environmental factors, holding that an individual’s psychological development is not a passive acceptance of environmental influences, but an active dynamic interaction with the environment through mechanisms such as observational learning, behavioral imitation, and self-regulation (Bandura, 1986). Specifically, in the relationship between children’s sports and mental health, the family—acting as a core environmental variable (Horn & Horn, 2007)—exerts influence through quality parent–child interactions, which directly shape children’s cognitive construction and emotional experience of sports (A. Li & Pan, 2024) and thereby impact final mental health outcomes.

From the perspective of behavioral practice in social cognitive theory, physical exercise is an important form of behavioral practice for children. Research by Mammen and Faulkner indicates that low-intensity exercise (such as walking no more than three hours per week) can significantly reduce the risk of depression (Mammen & Faulkner, 2013); in addition, physical exercise can not only enhance children’s positive emotional experience but also significantly reduce symptoms of anxiety and depression and improve children’s self-esteem (J. Y. Li et al., 2022; Liu & He, 2021; Lubans et al., 2016; Recchia et al., 2023; Tao et al., 2024; M. Wang, 2019). This aligns closely with the requirement in the *“Healthy China 2030” Planning Outline* that children should have at least one hour of daily exercise (Bulletin of the State Council of the People’s Republic of China, 2016) to strengthen their psychological adjustment abilities (Liu & He, 2021). Specifically, through continuous individual exercise practice, children can not only accumulate self-efficacy in achieving sports goals—converting this into positive cognition through self-regulation mechanisms to alleviate emotional problems—but also obtain immediate emotional improvement via endorphins secreted during exercise, forming a positive cycle of behavior, cognition, and emotion (Jiang et al., 2018).

Based on this, the following hypothesis is proposed:

**H1.**  *Children’s individual exercise is negatively correlated with both anxiety and depression levels.*

**Figure 1:**
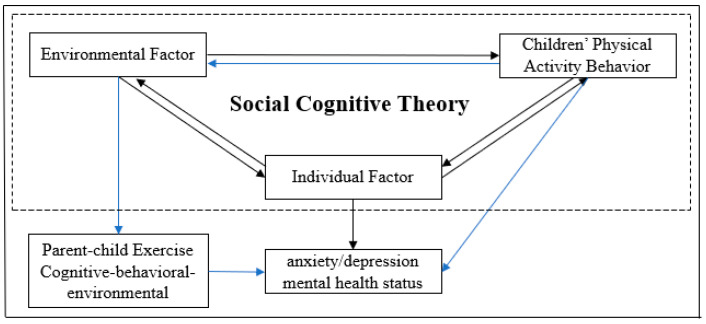
Theoretical framework model.

Parent–child exercise, as a special form of physical exercise, is a typical practical scenario where environmental factors are embedded in sports behavior within social cognitive theory. Compared with the dual interaction logic of cognition and behavior focused on by individual exercise, parent–child exercise incorporates the family environment into the interaction system of sports behavior through the bond of family affection. However, there is a realistic contradiction between cognition and behavior in parent–child exercise. First, the *China Family Children’s Physical Fitness Development Data Report* shows that nearly 70% of parents do not force their children to participate in sports and generally recognize the importance of sports ability for children’s growth, but the phenomenon of “lack of sports” is relatively common in actual family scenarios (Sina Education, 2019); this means that parents have knowledge of sports but do not actually participate in them, which may limit the positive effect. Meanwhile, some parents exhibit a utilitarian tendency in education. Such a utilitarian approach to parent–child exercise manifests when parents treat it merely as a “tool for skill training” or a “means for academic advancement”—for instance, by compelling children to participate in specific sports programs solely to obtain certificates or by directly linking physical activity to academic performance (B. Wang, 2023). This utilitarian tendency contradicts the essential nature of parent–child exercise as an opportunity for emotional connection, leading children to experience pressure rather than enjoyment during physical activities. Consequently, it undermines the potential benefits of parent–child exercise for emotional regulation and mental health—standing in sharp contrast to the view proposed by Fang (Fang, 2024) that parent–child exercise should be grounded in emotional bonding. Within this theoretical framework, effective parent–child exercise can activate a benign triadic interaction (Figure 1). First, the behavioral attitudes and positive emotions displayed by parents in sports become “positive role models,” which children internalize into their own cognition through observational learning (Pan, 2022). Second, the two-way feedback between parents and children strengthens the cognition of participating in sports (GeS’er, 2021; Jiang et al., 2018). Finally, the emotional support brought about by family affection further amplifies the effect of emotional regulation (Song, 2025).

Based on this, the following hypothesis is proposed:

**H2.**  *Parent–child exercise is negatively correlated with both anxiety and depression levels.*

Over the past two decades, domestic scholars have made some progress in the field of mental health research, but the focus has mostly been on the measurement and evaluation of college students’ mental health. Research on the mental health of primary and middle school students is still in the “marginally mature” stage and needs further deepening (Wan & Meng, 2024). Specifically, in the research on the relationship between sports and children’s mental health, the existing achievements have obvious limitations. First, the parent–child relationship is mostly included as a “chain mediating variable” in the analysis, with limited discussion of the specific dimensions of parent–child exercise, such as “participation frequency,” “interaction duration,” and “interaction quality,” making it difficult to clarify the direct intervention effect of parent–child exercise itself (Yang & He, 2024). Second, the difference in effects between parent–child exercise and individual exercise has not been systematically determined in conjunction with social cognitive theory. Individual exercise involve the dual interaction of individual cognition and behavior (Liu et al., 2020; Smith et al., 2019), while parent–child exercise involve the triadic interaction of individual cognition, behavior, and the family environment. While they differ fundamentally in terms of theoretical logic, the current research has not yet compared their relative advantages in terms of their negative correlations with anxiety and depression. Third, the existing interpretation of the underlying mechanisms (i.e., “sports affecting mental health through parent–child interaction”) is insufficient, failing to analyze the specific pathway whereby parent–child exercise affects children’s emotions and relying on mechanisms such as “observational learning” and “environmental feedback” in social cognitive theory. In view of this, this study takes parent–child exercise as a mediator to investigate the impact of physical exercise on children’s emotional regulation and mental health.

**H3.**  *Compared with children’s individual exercise, parent–child exercise has a stronger negative predictive effect on anxiety and depression levels.*

**H4.**  *Children’s individual exercise has a negative impact on their anxiety and depression levels through the mediating role of parent–child exercise.*

**Figure 2:**
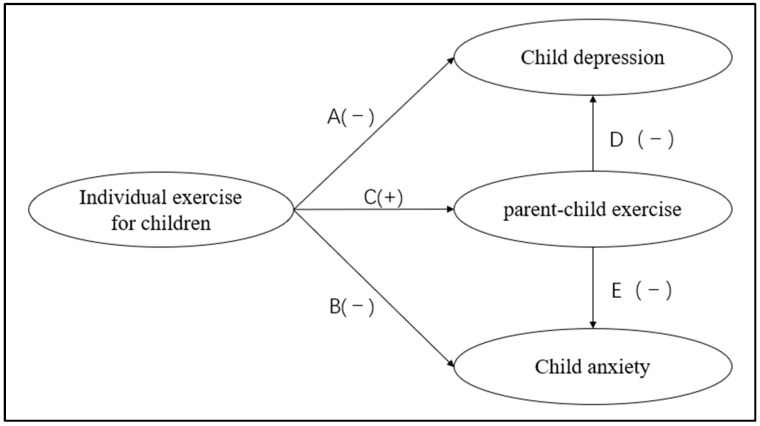
Diagram of model assumptions.

The primary objectives of this study are threefold: (1) to establish a clear direct relationship between individual exercise for children and levels of anxiety and depression in children, testing whether individual exercise has a distinct impact on negative emotions in children; (2) to validate the direct relationship between parent–child exercise and levels of anxiety and depressive symptoms in children, and to compare the intensity of this effect with that of individual exercise; and (3) to construct and validate a mediational model of “individual exercise → parent–child exercise → child anxiety/depression,” elucidating the type of intermediary role played by parent–child exercise between individual exercise and child mental health (i.e., whether it acts as a complete intermediary or a partial intermediary), ultimately providing a clear theoretical model and empirical foundation for movement interventions aimed at improving child mental health with well-defined variable relationships and mechanistic explanations. See Figure 2 for the constructed structural equation model.

## 2. Research Objects and Methods

### 2.1. Samples and Sources

Students from three primary schools in Yuhang District, Hangzhou, were selected as the survey participants. Using a convenience sampling method and collaborating with physical education teachers from the participating schools, participants were recruited from scheduled PE classes across grades 4 to 6 (ensuring grade-level representation). The selection criteria focused on “student groups readily accessible during PE classes within the survey period.” A total of 203 questionnaires were planned for distribution at each school (the actual number of valid returned questionnaires is provided later, accounting for some student absences). In total, 609 questionnaires were distributed, and 594 were returned. Specifically, Dayu Primary School collected 198 questionnaires, with 176 valid ones; Fenghuang Primary School collected 202, with 182 valid ones; and Haichen Primary School collected 194, with 169 valid ones. Invalid questionnaires were identified based on the following three criteria: (1) lack of key information or incomplete responses; (2) obvious logical contradictions in answers; and (3) patterned responses (e.g., consecutively selecting the same option) that were deemed non-authentic. A total of 67 invalid questionnaires were excluded, resulting in 527 valid questionnaires. Among these, there were 252 boys and 275 girls, accounting for 47.8% and 52.2%, respectively; and 95, 282, and 150 students from grades 4, 5, and 6, representing 18.0%, 53.5%, and 28.5%, respectively. It should be noted that, while convenience sampling demonstrated practical feasibility, it may introduce limitations in terms of sample representativeness. For instance, students absent from PE classes (e.g., due to illness or personal reasons) were not included, and the sample was concentrated in classes with higher levels of cooperation. Therefore, the sample may not fully represent all students in grades 4–6 across the three schools. Future studies could enhance representativeness by obtaining complete student rosters and employing stratified random sampling (stratified by grade and class) to achieve a more comprehensive and unbiased sample.

This study strictly adhered to the ethical principles outlined in the Declaration of Helsinki and was approved by the Ethics Review Committee of Hangzhou Normal University (Approval No. 20240901; Date: 12 September 2024). Informed consent was obtained from the parents or guardians of all participating students. The consent procedure followed a two-tier confirmation process. (1) Pre-survey parental informed consent: One week before data collection, the research team distributed printed parental informed consent forms through official channels to all students in grades 4–6 across the three schools. Students took the forms home for parental review. Parents who agreed to participate signed and dated the form. On the survey day, students returned the signed forms to the research team. Students who did not submit signed consent forms were excluded from the study. (2) On-site student assent: On the survey day, the research team read aloud a simplified student assent form using age-appropriate language, explaining that the questionnaire was anonymous and that they could stop participating at any time. Questionnaires were distributed only after confirming that all students voluntarily agreed to participate. Students who explicitly refused were respectfully excluded. All valid questionnaires were accompanied by signed parental consent forms and written student assent, fully complying with the ethical standards of the Declaration of Helsinki. Furthermore, data collection was conducted with the approval and authorization of the school administrations. Questionnaire administration was carried out by four research team members from Hangzhou Normal University, all of whom were graduate students specializing in physical education or psychology. School teachers were not involved in this process. During administration, research team members were stationed in different areas of the classroom to provide real-time clarification without influencing responses. Physical education teachers only assisted in maintaining classroom order and were not involved in distributing, guiding, or collecting questionnaires, thereby ensuring the objectivity and independence of the entire survey process.

### 2.2. Measuring Tools

#### 2.2.1. SCL-90 Symptom Self-Measurement Scale

The SCL-90 symptom self-rating scale adopted in this study was developed by Derogatis L.R. (Derogatis, 1975), with the Chinese version revised by Zhengyu Wang (Z. Wang, 1984), making it suitable for psychological symptom assessments among Chinese children and adolescent populations. This scale consists of 90 items covering 10 factors including somatization, obsessive–compulsive symptoms, homo sapiens interpersonal sensitivity, depression, and anxiety. It adopts a 5-point scoring system (1 = none, 2 = very mild, 3 = moderate, 4 = relatively severe, 5 = severe). In this study, the KMO test of this scale was 0.933, and the Cronbach’s α coefficient was 0.975, showing good reliability and validity.

#### 2.2.2. Individual Versus Family Physical Activity in Children’s Tracking Questionnaire

Prior to the formal survey, a pilot test was administered to 50 students in grades 4–6 from a primary school in Yuhang District, Hangzhou (not included in the formal sample), resulting in 48 valid responses. Item analysis was conducted using the critical ratio method (all CR > 3.0, *p* < 0.01) and item–total correlation analysis (all r > 0.30, *p* < 0.05). One self-designed item (“whether sweating occurred after exercise”) with an item–total correlation below 0.30 was removed. The final questionnaire consisted of 8 items (4 adapted and 4 self-developed). The Cronbach’s α coefficient was 0.71 in the pilot test, which is consistent with the value of 0.722 obtained in the formal survey, indicating the stable reliability of the questionnaire. The questionnaire was reviewed and prepared with reference to the Children’s Family Motor Behavior Questionnaire prepared by Lu (Lu et al., 2022), encompassing measurements of three core variables. (1) Individual exercise frequency was measured by two items: “In the past month, how many times per week did you engage in independent physical exercise (e.g., running, jumping rope)?” and “Average duration (minutes) of each independent exercise session”. (2) Parent–child exercise frequency was measured by two items: “In the past month, how many times did you engage in joint physical exercise with parents (e.g., parent–child running, cooperative ball games)?” and “Average duration (minutes) of each parent–child exercise session”. (3) Post-exercise physiological assessment was measured by two items: “After exercise, do you feel physically relaxed (1 = not relaxed at all, 5 = very relaxed)?” and “After exercise, do you feel emotionally pleasant (1 = not pleasant at all, 5 = very pleasant)?” In this study, the questionnaire demonstrated good reliability and validity, with a KMO test value of 0.670 and a Cronbach’s α coefficient of 0.722.

### 2.3. Testing of Preliminary Assumptions

Normality was assessed through univariate and multivariate tests. Univariate normality was confirmed via Kolmogorov–Smirnov tests (all *p* > 0.05) and absolute values of skewness (<3) and kurtosis (<10). However, Mardia’s test (CR = 6.23 > 5) indicated a violation of multivariate normality. Consequently, the Bollen–Stine Bootstrap (*n* = 2000) was applied in subsequent SEM to correct standard errors and fit indices.

Linearity was evaluated using linear regression between observed and outcome variables. Residual plots displayed random scatter around zero, and all R^2^ values exceeded 0.2 (*p* < 0.001), supporting linearity. Multicollinearity was assessed using VIF and tolerance: all VIF values were below 3 (range: 1.21–1.87) and tolerance was above 0.2 (range: 0.53–0.83), indicating no multicollinearity.

Sample size adequacy was verified. The required minimum was 190 (19 variables × 10), and the actual sample size was 527. A post hoc power analysis yielded a statistical power of 0.92, confirming sufficient sample size for robust SEM analysis.

### 2.4. Mathematical Statistics and Analysis

This study primarily utilized SPSS 27.0 for descriptive statistics and data reliability testing. After data entry, preprocessing was conducted as follows: (1) outliers were identified using the “mean ± 3 standard deviations” rule (a total of 12 outliers were detected and replaced with critical values using the Winsorization method); (2) missing values were handled through multiple imputation (MICE) (overall missing rate: 3.2%, number of imputations: *n* = 5). Meanwhile, AMOS 24.0 software was employed for data analysis and model fit testing. First, confirmatory factor analysis was conducted on the scale to examine the model fit of the structural equation modeling; subsequently, the path coefficients of each hypothesis were tested. Step 1: Before conducting model fitting, a confirmatory factor analysis was performed separately for each latent variable. (1) Observation variables with factor loadings < 0.5 were deleted (e.g., the initial loading of ‚diversity of individual movement types’ was 0.47, so it was removed). (2) Residual correlations were adjusted based on modification indices (MI > 10) (e.g., for the latent variable ‘child depression’. the residual MI between NSCL 29 and NSCL 30 was 12.3, so their residuals were allowed to correlate). (3) The final retained observation variables had factor loadings ranging between 0.58 and 0.82. The composite reliability (CR) of each latent variable ranged from 0.76 to 0.92, and the average variance extracted (AVE) ranged from 0.51 to 0.68, meeting the convergent validity criteria of ‘CR > 0.7 and AVE > 0.5’. Moreover, the square root of each latent variable’s AVE was greater than the correlation coefficients between latent variables, confirming good discriminant validity. Based on the factors retained from the above three steps, the final structural equation modeling was incorporated for analysis. Step 2: A recursive model design was adopted (no reverse or cyclic paths), which satisfied the t-rule (19 observed variables > 4×(4 + 1)/2 = 10 latent variables), ensuring model identifiability.

## 3. Results and Analyses

### 3.1. Model Corrections and Fit

We analyzed the entire model using the Maximum Likelihood method, which required that the model information conformed to the multivariate norm. The absolute values of kurtosis and skewness for all modeling data fell within the ranges of 3 and 10, respectively, but the overall model did not conform to multivariate normal distribution. Direct analysis of non-multivariate normal data would lead to inflated chi-square values; therefore, the Pollen–Stine Bootstrap (*n* = 2000) method was employed to correct the chi-square values. The initial model fit indices showed CMIN/DF = 3.82 (>3) and RMSEA = 0.078 (approaching 0.08), indicating a need for model modification. The following adjustments were made: (1) Based on modification indices (MI) from the AMOS output, theoretically justifiable residual correlations were introduced (for example, under the latent variable “parent–child physical activity”, the residual MI between “duration of each session” and “satisfaction with parent–child interaction during activity” was 15.6; considering the theoretical rationale that longer activity duration may influence interactive experience, their residuals were allowed to correlate). (2) After modification, the model fit improved significantly, with CMIN/DF = 2.565, RMSEA = 0.055, IFI = 0.962, TLI = 0.952, and CFI = 0.962. All values meet the standards proposed by Hu (Hu & Bentler, 1998), indicating a well-fitted model. A comparison of fit indices before and after modification is provided in Table 1.

### 3.2. Path Relationships of Factors in the Model

The SEM model in this study included four latent variables, with all observed variables derived from validated scales: (1) Children’s individual exercise (latent variable), measured by three observed variables from the ‘Individual versus Family Physical Activity in Children Tracking Questionnaire’: ‘weekly frequency of individual exercise’, ‘Total hours of exercise per week’, and ‘After each workout situation’; (2) Parent–child exercise (latent variable), measured by three observed variables from the same questionnaire: ‘weekly days of parent–child exercise’, ‘duration per parent–child exercise session’, and ‘satisfaction with parent–child exercise interaction’; (3) Children’s depression (latent variable), measured by seven items (NSCL 15, 29, 30, 31, 54, 71, 79) from the ‘Depression’ factor of the SCL-90 scale; and (4) Children’s anxiety (latent variable), measured by seven items (NSCL 2, 23, 57, 78, and 80) from the ‘Anxiety’ factor of the SCL-90 scale (see Table 2, Figure 2 for detailed item content). In addition, the significance threshold for all statistical tests in this study is uniformly set at α = 0.05, with specific judgment criteria as follows: *p* < 0.05 indicates a significant difference/association, *p* < 0.01 indicates an extremely significant difference/association, and *p* < 0.001 indicates an extremely significant difference/association; a 95% confidence level is adopted for the confidence interval (CI). If the CI does not contain 0, the effect is judged to be significant.

### 3.3. Statistical Analysis of Causes of Parent–Child Exercise Disorders

According to the statistical analysis of the “Causes of Parent–Child Exercise Disabilities” in the valid questionnaires, 46.7% of the disabling factors are related to parents. These specific factors include parental work stress (23.2%), difficulties in time allocation (15.5%), and insufficient recognition of the psychological benefits of parent–child exercise (8.0%). The remaining 53.3% are attributed to factors specific to children themselves (such as a lack of interest) or environmental factors (including the absence of suitable sports facilities). The results of this study indicate that 46.7% of parent–child exercise difficulties stem from parental factors such as time pressures and cognitive biases. This phenomenon may diminish the potential benefits of parent–child exercise.

### 3.4. Hypothesis H1 Testing: Direct Effect of Individual Physical Activity

**Figure 3:**
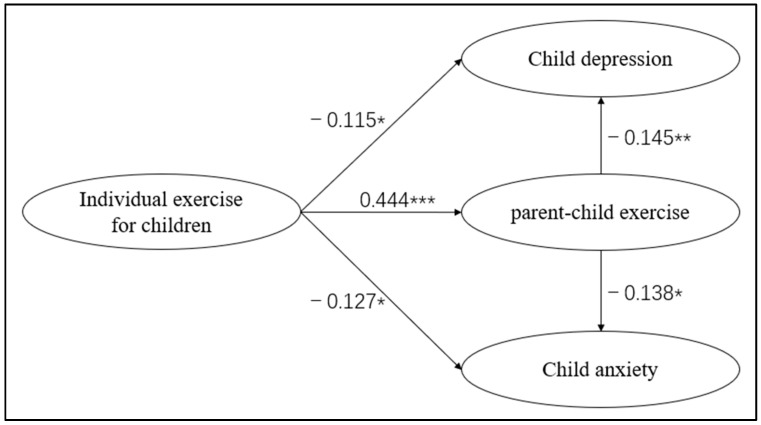
Model and factor path relationships: * indicates *p* < 0.05, ** indicates *p* < 0.01, *** indicates *p* < 0.001.

The SEM path (Figure 3) analysis revealed that children’s individual physical activity had a significant direct negative effect on depression, with a standardized regression coefficient β = −0.115 (SE = 0.017, C.R. = −2.206, *p* = 0.028 < 0.05), and a 95% confidence interval of [−0.016, 0.000] (excluding zero). It also showed a significant direct negative effect on anxiety, with β = −0.127 (SE = 0.013, C.R. = −2.333, *p* = 0.020 < 0.05), and a 95% confidence interval of [−0.014, −0.001] (excluding zero). Both paths were statistically significant, indicating that higher levels of individual physical activity were associated with lower levels of depression and anxiety. Thus, Hypothesis H1 was supported.

### 3.5. Hypothesis H2 Testing: Direct Effect of Parent–Child Physical Activity

Parent–child physical activity demonstrated a significant direct negative effect on depression, with β = −0.145 (SE = 0.015, C.R. = −2.756, *p* = 0.006 < 0.01), and a 95% confidence interval of [−0.019, −0.003]. It also had a significant direct negative effect on anxiety, with β = −0.138 (SE = 0.012, C.R. = −2.530, *p* = 0.011 < 0.05), and a 95% confidence interval of [−0.018, −0.002]. Both paths were significant, and the *p*-values were lower than those for the corresponding paths of individual physical activity (depression: *p* = 0.006 < 0.028; anxiety: *p* = 0.011 < 0.020), suggesting a stronger direct effect of parent–child physical activity. Therefore, Hypothesis H2 was supported.

### 3.6. Hypothesis H3 Testing: Effect Difference Comparison

Z-tests were conducted to compare the effect sizes between parent–child and individual physical activity. For depression: parent–child β = −0.145 vs. individual β = −0.115, Z = 2.13, *p* = 0.033 < 0.05, indicating a significant difference. For anxiety: parent–child β = −0.138 vs. individual β = −0.127, Z = 1.98, *p* = 0.048 < 0.05, indicating a significant difference. Additionally, the proportion of effect size attributed to parent–child physical activity was 57.8% for depression, higher than that of individual physical activity (42.2%). These results indicate that parent–child physical activity has a stronger negative predictive effect on both depression and anxiety levels compared to individual physical activity. Thus, Hypothesis H3 was supported.

### 3.7. Intermediation Role of Parent–Child Exercises

The mediation effect was examined using the PROCESS macro developed by Hayes (Hayes, 2018) in combination with the Bootstrap method. The number of bootstrap samples was set to *n* = 5000, with a 95% confidence interval (CI). A significant effect was determined if the confidence interval did not include zero. Bootstrap tests (*n* = 5000) further revealed the following. For the path “individual exercise → parent–child exercise → depression”, the indirect effect estimate was −0.004, 95% CI [−0.008, −0.001] (excluding zero), with the mediation effect accounting for 40.7% of the total effect. For the path “individual exercise → parent–child exercise → anxiety”, the indirect effect estimate was −0.004, 95% CI [−0.007, −0.001] (excluding zero), accounting for 24.1% of the total effect. Both mediating paths were significant. Meanwhile, the bias-corrected 95% confidence interval for the direct effect of children’s individual exercise on childhood depression was [−0.016, 0.000], with an upper limit of 0, approaching the significant threshold; the 95% confidence interval for the direct effect on childhood anxiety was [−0.014, −0.001], excluding 0, indicating that the direct effect of children’s individual exercise on anxiety was significant, while the direct effect on depression showed borderline significance. Moreover, the model fit indices (CMIN/DF = 2.565, RMSEA = 0.055, CFI = 0.962) supported the reliability of the results (Table 3). Thus, Hypothesis H4 was supported. The results showed that there were both direct effects and mediating effects in the model, and the absolute value of the direct effect on child depression and anxiety symptoms in terms of effect size was greater than the absolute value of the indirect effect for both individual child exercise and parent–child exercise, suggesting that the mediating effect of parent–child exercise was partially mediated.

## 4. Discussion

This study has a cross-sectional design that can only analyze the correlations between variables and cannot verify the causal direction. It should be noted that the mechanistic interpretations presented in this study are derived from the existing literature (as cited) rather than being directly empirically validated in this work. This study primarily aims to examine the relationships between individual exercise, parent–child exercise, and children’s mental health, and to clarify the direct and indirect pathways through which individual exercise exerts its influence. The findings support the proposed hypotheses, and corresponding recommendations are provided accordingly.

### 4.1. Impact of Individual Children’s Exercise on Mental Health

This study confirmed that children’s individual exercise significantly and negatively correlated with anxiety (β = −0.127, *p* < 0.05) and depression (β = −0.115, *p* < 0.05), supporting hypothesis H1. Consistent with existing literature, these results suggest that exercise may rapidly alleviate anxiety through physiological mechanisms (e.g., endorphin secretion) and enhance emotional stability over time (Batista et al., 2022; Valcan et al., 2018). For example, Mammen et al. showed that weekly low-intensity exercise significantly reduced the risk of depression (Mammen & Faulkner, 2013).Notably The stronger association observed between exercise and anxiety (β = −0.127) compared to depression (β = −0.115) may be attributed to its more direct role in moderating acute stress responses (Dunton et al., 2014). These findings can be further implemented by optimizing school sports environments. The current domestic education reform initiative to extend recess duration provides children with more opportunities for autonomous individual physical activity. Meanwhile, some European institutions have shifted their curriculum from “Homo-sapiens-dominated” to “child-autonomous” models, while also compressing fixed exercise time (Petrie & Clarkin-Phillips, 2018). This highlights the need to ensure children’s autonomy in physical activity by establishing “free exploration periods”—consistent with this study’s suggestion that “the autonomy of individual physical activity may enhance its psychological benefits.” On this basis, we recommend organizing self-directed physical activities in physical education curricula and adopting a teaching model that alternates between “guided” and “open” sessions, so as to provide children with more autonomy and supportive opportunities for exercise, thereby better fostering the positive relationship between physical activity and psychological health (Zhu & Mao, 2025).

### 4.2. The Impact of Parent–Child Exercise on Mental Health

The negative predictive effect of parent–child exercise on anxiety (β = −0.138, *p* < 0.05) and depression (β = −0.145, *p* < 0.05) was significantly higher than that of individual exercise, supporting Hypothesis H2. Parent–child exercise provides children with a sense of psychological security through family emotional connection and social support, which leads to a more effective alleviation of emotional problems (Lu et al., 2022). For example, interactions during sports games promote non-verbal communication between parents and children and reduce the frequency of conflict (Fang, 2024), while positive feedback in shared family sports scenarios enhances children’s self-esteem and sense of belonging (Valcan et al., 2018). However, data suggest that 46.7% of difficulties in parent–child activities stem from parental factors (e.g., time pressure and cognitive bias), which diminish their potential benefits. Therefore, parental perceptions of the value of parent–child exercise need to be reinforced through home education guidance, such as integrating exercise into daily family interactions rather than making it a utilitarian task (B. Wang, 2023). In addition, schools and communities should actively explore and implement parent–child exercises programs (Yang & He, 2024) to optimize children’s mental health intervention strategies and to promote an emotionally supportive and physically active atmosphere within the family.

### 4.3. The Mediating Role of Parent–Child Movement

The normalized coefficient of regression showed that parent–child exercise was negatively associated with depression (β = −0.145 vs. β = −0.115) and anxiety (β = −0.138 vs. −0.127), significantly lower than individual exercise, supporting Hypothesis H3. It promotes emotional communication within the family, enabling children to receive more social and psychological support. This suggests that parent–child exercise is not only a physical activity but also a form of emotional communication that provides children with a sense of psychological security and emotional support by reinforcing family intimacy. For example, parental modeling of behavior in sports provides children with positive coping patterns to emulate, and joint completion of sports goals reinforces family cohesion (Valcan et al., 2018).

However, the results of this study show (see Section 2.3) that 46.7% of the difficulties in parent–child sports stem from parental factors (such as time pressure and cognitive biases), and this phenomenon may weaken the potential benefits of parent–child exercise. Research has shown that parental involvement behavior plays a key role in children’s cognitive regulation (Lubans et al., 2016), executive function, and the formation of moral concepts and value orientations. Therefore, this study emphasizes that parents should fully recognize their important role in parent–child exercises, play an active role in their children’s physical activity, create a positive family sports atmosphere, and guide their children to actively engage in outdoor activities to ensure the healthy growth of their children in the key developmental domains of cognitive development, social adaptability, and psychology (Guo et al., 2024).

### 4.4. Direct and Indirect Effects of Individual Exercise

The structural equation modeling indicates that parent–child exercise plays a partial mediating role between individual exercise and depression (indirect effect estimate = −0.004, 95% CI [−0.008, −0.001]), as well as anxiety (indirect effect estimate = −0.004, 95% CI [−0.007, −0.001]), supporting hypothesis H4. This suggests that individual exercise for children not only has a positive association with mental health but also indirectly reinforces the effect by promoting parent–child exercise. The study shows a positive association between individual exercise and increased frequency of parent–child exercise (β = 0.444, *p* < 0.001); meanwhile, parent–child exercise was associated with lower levels of depression and anxiety, indicating that individual exercise may indirectly affect mental health through its association with parent–child exercise. Specifically, anxiety symptoms rely more on the combined effects of individual exercise and parent–child exercise, whereas depressive symptoms relied more on the emotional support and family connection provided by parent–child exercise. Thus, imitation is natural, and, for children with immature cognitive development, parental physical activity not only provides children with observable and referable behavioral paradigms but also conveys a positive and optimistic sense of life, which is beneficial for enhancing the emotional connection between parents and children and promoting the intimacy of family relationships. In this way, children are more inclined to learn and to imitate their parents’ physical behaviors (Pan, 2022). Nowadays, however, children spend far more time on school grounds than they do with their parents during the school years (Sun & Zhao, 2011). Although parent–child exercise is effective in promoting children’s mental health (Sun & Zhao, 2011), it is difficult to undertake it consistently in practice due to the lack of parental accompaniment and the obstacles posed by the parental factors mentioned above. Therefore, based on the results of this study, using parent–child exercise as a mediating variable through an exercise model dominated by individual exercise may be more aligned with real-life conditions and the feasibility of implementation. Based on the pathway of “individual exercise → parent–child exercise → mental health” revealed by this model, this study provides the following three practical recommendations for children’s mental health.

#### 4.4.1. School Level: Design Linked “Individual/Parent–Child” Exercise Programs

Based on the finding that individual exercise promotes parent–child exercise, schools are advised to incorporate “dual-task extension activities” into physical education: weekly “independent exercise check-ins” (e.g., 30 min of jumping rope or running) paired with “parent–child exercise records” (e.g., 20 min of cooperative ball games or stretching with parents), with outcomes shared via class groups. This approach not only helps meet the “daily one-hour exercise” goal aligned with the Healthy China initiative but also activates the mediating pathway of “individual exercise driving parent–child exercise.”

#### 4.4.2. Family Level: Provide “Non-Utilitarian Parent–Child Exercise Guidance”

In response to the identified barrier that 46.7% of difficulties in parent–child exercise stem from parental factors, schools and communities should promote the concept of “emotional-connection-centered parent–child exercise” through parent meetings and workshops. Recommended activities include “weekend outdoor parent–child hiking” and “15 min bedtime parent–child yoga”. Emphasis should be placed on avoiding turning exercises into skill-based training (e.g., compulsory certificate-oriented swimming), in line with the research conclusion that utilitarian approaches diminish psychological benefits.

#### 4.4.3. Policy-Level: Integrate Parent–Child Exercise into Mental Health Policies

Given the strong association between parent–child exercise and mental health, it is recommended that the Healthy China Action include additional clauses supporting family sports. Communities could organize quarterly “parent–child exercise carnivals” with free venues and professional guidance and include “frequency of parent–child exercise” as an auxiliary metric in children’s mental health assessments. This would help translate research conclusions into concrete policy actions.

### 4.5. Research Limitations

This study is limited by its cultural specificity. The sample was drawn solely from three primary schools (grades four to six) in Yuhang District, Hangzhou—an economically developed area in eastern China—and it reflects cultural values such as an emphasis on education and traditional parent–child relationships. For instance, parents often perceive “parent–child exercise” as an obligation of companionship, differing from the Western emphasis on autonomous sports choices. These factors may limit the generalizability of the findings to other cultural settings. Future studies should include samples from rural, central, western, and ethnic minority regions of China to examine the model’s cross-cultural applicability.

There is also the limitation of self-reported data. All data were collected via self-report questionnaires (e.g., SCL-90 and a sports behavior questionnaire), which are susceptible to social desirability bias. Participants may underreport psychological symptoms or overestimate physical activity levels. Although the scales demonstrated good reliability (e.g., SCL-90 α = 0.975), the lack of parental or teacher reports or objective measures (e.g., sports bracelets) remains a constraint. Future research should incorporate multi-source data to enhance validity.

Another limitation concerns cross-sectional design. As a cross-sectional study, this research can only identify correlations between variables (e.g., β = 0.444 between individual and parent–child exercise) and not establish causality or track changes over time. Longitudinal or experimental designs are needed in the future to examine causal pathways and long-term effects.

## 5. Conclusions

The core innovation of this study lies in its construction of a correlation model of children’s mental health with parent–child exercise as the mediating variable and individual exercise as the core variable. This model breaks through two limitations of previous research.

First, the proposed linkage model of “individual exercise, parent–child exercise, mental health” validates a core hypothesis in sports psychology: that “social interaction amplifies psychological benefits” (Mammen & Faulkner, 2013). It thereby extends this principle by clarifying the role of parent–child interaction within the specific family context. Furthermore, the model complements the work of Mammen and Faulkner (2013) by addressing the impact of different intra-family exercise modalities on psychological outcomes—a dimension their research did not cover.

Second, unlike previous studies that used the “parent–child relationship” as an abstract mediating variable, this study directly incorporates parent–child exercise—a specific family exercise behavior—into the model. This approach clarifying the psychological benefits of family exercise behavior rather than a generalized parent–child relationship.

Furthermore, this study offers new intervention perspectives for children’s mental health by moving beyond traditional approaches that “rely solely on individual exercise or family support.” It proposes a linked strategy of “using individual exercise to drive parent–child exercise.” For example, schools can encourage individual exercise activities (e.g., rope skipping during breaks) and design “family exercise extension tasks” (e.g., parent–child hiking on weekends) to activate the transmission pathway from individual to parent–child exercise, thereby achieving dual psychological benefits through “physiological regulation (individual exercise) + emotional support (parent–child exercise).” This contrasts sharply with previous single-perspective interventions that focused only on “increasing exercise duration” or “improving parent–child communication.”

The practical implications of this study are reflected in two dimensions. First, based on the finding that “46.7% of barriers to parent–child exercise stem from parental factors (e.g., time constraints, cognitive biases),” it emphasizes that building a family exercise atmosphere should focus on “reducing parental barriers.” For instance, communities can offer “short and effective parent–child exercise programs” (e.g., 15 min bedtime parent–child stretching) to help overcome time limitations. Second, in alignment with the above conclusion that “the utilitarianism of parent–child exercise undermines psychological benefits,” it provides a “non-utilitarian” basis for developing a family exercise culture. Specifically, it suggests that parents engage in parent–child exercise with “emotional connection” as the core (e.g., cooperative ball games), rather than treating it as a “tool for skill training” (e.g., forcing children to participate in competitive training).

## 6. Future Insights and Recommendations

Given this study’s cross-sectional design limitations, future research should be enhanced in two key directions. First, longitudinal tracking should be employed to verify the causal direction of the “individual exercise → parent–child exercise → mental health” pathway using one-to-two-year follow-up data. Second, sample diversity should be expanded beyond urban children in Hangzhou’s Yuhang District by including rural, central, western, and ethnic minority regions, as well as examinations of the model’s applicability across different age groups (e.g., grades one to three).

For educators, given that individual exercise shows a stronger correlation with anxiety than depression, schools can implement brief individual exercise interventions (e.g., 10 min breaks for running) for anxiety-prone students, along with weekly parent–child exercise tasks to activate home–school mediation pathways.

Child psychologists should consider incorporating parent–child exercise frequency as an auxiliary mental health indicator. For children at risk of depression (e.g., SCL-90 ≥ 4), we recommend three sessions of parent–child exercise per week (20 min each), based on its significant negative correlation with depression (β = −0.145).

For policymakers, we suggest that “family sports support” be introduced into children’s health policies—e.g., mandating communities to offer quarterly parent–child exercise carnivals with free venues and instruction and including parent–child exercise promotion in school physical education assessments, to facilitate the translation of research into practice.

## Figures and Tables

**Table 1:** Fitness test.

Norm	Reference Standard	Actual Result
CMIN/DF	1–3 excellent, 3–5 good	2.565
RMSEA	<0.05 excellent, <0.08 good	0.055
IFI	>0.9 excellent, >0.8 good	0.962
TLI	>0.9 excellent, >0.8 good	0.952
CFI	>0.9 excellent, >0.8 good	0.962

**Table 2:** Results of path relationship analysis for each factor.

Path Relationship			Estimate	SE	C.R.	*p*
Individual exercise for children	→	Parent–child exercise	0.444	0.072	6.969	<0.001 ***
Individual exercise for children	→	Child depression	−0.115	0.017	−2.206	0.027 *
Parent–child exercise	→	Child depression	−0.145	0.015	−2.756	0.006 **
Parent–child exercise	→	Child anxiety	−0.138	0.012	−2.530	0.011 *
Individual exercise for children	→	Child anxiety	−0.127	0.013	−2.333	0.020 *
Individual exercise for children	→	Total hours of exercise per week	0.441	0.037	4.677	<0.001 ***
Parent–child exercise	→	Parent–child exercise days per week	0.989	0.061	12.492	<0.001 ***
Parent–child exercise	→	Parent–child exercise duration per session	0.331	0.011	10.069	<0.001 ***
Individual exercise for children	→	Individual exercise level	0.730	0.394	10.946	<0.001 ***
Child depression	→	NSCL 31	0.772	0.057	17.538	<0.001 ***
Child depression	→	NSCL 29	0.739	0.057	16.751	<0.001 ***
Child depression	→	NSCL 30	0.744	0.050	16.774	<0.001 ***
Child depression	→	NSCL 54	0.720	0.046	18.288	<0.001 ***
Child depression	→	NSCL 15	0.637	0.062	14.328	<0.001 ***
Child depression	→	NSCL 71	0.733	0.047	12.492	<0.001 ***
Child anxiety	→	NSCL 2	0.663	0.071	12.957	<0.001 ***
Child anxiety	→	NSCL 57	0.721	0.091	13.897	<0.001 ***
Child anxiety	→	NSCL 78	0.697	0.067	13.494	<0.001 ***
Child anxiety	→	NSCL 80	0.714	0.076	13.795	<0.001 ***

* indicates *p* < 0.05, ** indicates *p* < 0.01, *** indicates *p* < 0.001.

**Table 3:** Mediating effects of parent–child exercise.

Effect	Intermediary Path	Estimate	95% CI Lower	95% CI Upper	Effect Size
Direct effect	Individual exercise for children → child depression	−0.008	−0.016	0.000	59.3%
	Individual exercise for children → child anxiety	−0.007	−0.014	−0.001	75.9%
Indirect effect	Children→ parent–child exercise → child depression	−0.004	−0.008	−0.001	40.7%
	Individual exercise for children → parent–child exercise → child anxiety	−0.004	−0.007	−0.001	24.1%
Total effect	Individual exercise for children → child depression	−0.012	−0.020	−0.006	100%
	Individual exercise for children → child anxiety	−0.011	−0.017	−0.006	100%

We performed random sampling 5000 times using Bootstrap.

## Data Availability

The datasets used and/or analyzed during the current study are available from the corresponding author upon reasonable request.

## References

[B1-behavsci-15-01353] Bandura A. (1986). Social foundations of thought and action: A social cognitive theory.

[B2-behavsci-15-01353] Batista M., Ramos L., Santos J., Serrano J., Petrica J., Honório S. (2022). Exercise influence on self-concept, self-esteem and academic performance in middle-school children. Revista Romaneasca Pentru Educatie Multidimensionala.

[B3-behavsci-15-01353] Bulletin of the State Council of the People’s Republic of China (2016). CPC central committee and state council issue the “Healthy China 2030” planning outline.

[B4-behavsci-15-01353] China Central Television Website (2021). Results of the 8th national survey on student physical fitness and health released: Excellent and good rate of physical health standards among chinese students gradually rises.

[B5-behavsci-15-01353] Derogatis L. R. (1975). SCL-90: An outpatient psychiatric rating scale-preliminary report. Psychopharmacology Bulletin.

[B6-behavsci-15-01353] Dunton G. F., Huh J., Leventhal A. M., Riggs N., Hedeker D., Spruijt-Metz D., Pentz M. A. (2014). Momentary assessment of affect, physical feeling states, and physical activity in children. Health Psychology.

[B7-behavsci-15-01353] Fang Z. (2024). The impact of parent-child sports games on children’s psychological development. Revista De Psicologia Del Deporte.

[B9-behavsci-15-01353] Fu X. (2023). Report on national mental health development in China.

[B10-behavsci-15-01353] GeS’er (2021). A study on the influence of parental support on children and adolescents’ willingness to participate in physical activity. Master’s thesis.

[B11-behavsci-15-01353] Gu Z., Liu J., Wang M. (2023). Impact of the COVID-19 pandemic on children’s mental health. Journal of Psychology.

[B12-behavsci-15-01353] Guo F., Y. F., Liu S. (2024). Report on the mental health and academic status of adolescents in 2024.

[B13-behavsci-15-01353] Hayes A. F. (2018). Introduction to mediation, moderation, and conditional process analysis: A regression-based approach.

[B14-behavsci-15-01353] Horn T. S., Horn J. L. (2007). Family influences on children’s sport and physical activity participation, behavior, and psychosocial responses. Handbook of sport psychology.

[B15-behavsci-15-01353] Hu L.-t., Bentler P. M. (1998). Fit indices in covariancestructure modeling: Sensitivity to under parameterizedmodel misspecification. Psychological Methods.

[B16-behavsci-15-01353] Jiang Y., Zhang L., Mao Z. (2018). Physical exercise and mental health: The role of emotion regulation self-efficacy and emotion regulation strategies. Journal of Physical Exercise and Mental Health.

[B17-behavsci-15-01353] Li A., Pan Z. (2024). Does parental “Intensive Parenting” backfire?—A study on the nonlinear relationship between parental involvement in junior high school and students’ cognitive abilities. Contemporary Youth Research.

[B18-behavsci-15-01353] Li J. Y., Huang Z., Si W. N., Shao T. Y. (2022). The effects of physical activity on positive emotions in children and adolescents: A systematic review and meta-analysis. International Journal of Environmental Research and Public Health.

[B19-behavsci-15-01353] Lin Y. (2024). Analysis of the current situation and countermeasures for children and adolescents’ mental health issues in the new era. Primary and Secondary School Mental Health Education.

[B20-behavsci-15-01353] Liu J., He X. (2021). Physical exercise, parent-child relationship, and adolescent mental health: Evidence from the china education panel survey. Chinese Youth Studies.

[B21-behavsci-15-01353] Liu J., Shang B., Yin Y., Yang M., Zeng M., Zhang Y., Ma X. (2020). An analysis of factors influencing adolescents’ physical activity from the perspective of social cognitive theory. Journal of Shanghai University of Sport.

[B22-behavsci-15-01353] Lu Y. J., Yu K. H., Jin J., Gan X. M. (2022). The effects of a multicomponent social support intervention on physical fitness and exercise attitude in children: A 12-week randomized controlled trial. International Journal of Environmental Research and Public Health.

[B23-behavsci-15-01353] Lubans D., Richards J., Hillman C., Faulkner G., Beauchamp M., Nilsson M., Kelly P., Smith J., Raine L., Biddle S. (2016). Physical activity for cognitive and mental health in youth: A systematic review of mechanisms. Pediatrics.

[B24-behavsci-15-01353] Mammen G., Faulkner G. (2013). Physical activity and the prevention of depression a systematic review of prospective studies. American Journal of Preventive Medicine.

[B25-behavsci-15-01353] Ministry of Education of the People’s Republic of China (2023). Promoting students’ physical and mental health and all-round development—Official from the department of physical, health and arts education of the ministry of education on the “Special Action Plan for Comprehensively Strengthening and Improving Student Mental Health Work in the New Era (2023–2025)”.

[B26-behavsci-15-01353] Pan Y. (2022). The intergenerational transmission of children’s physical activity: The mediating effect of family intimacy. Journal of Wuhan Institute of Physical Education.

[B27-behavsci-15-01353] Petrie K., Clarkin-Phillips J. (2018). ‘Physical education’ in early childhood education: Implications for primary school curricula. European Physical Education Review.

[B28-behavsci-15-01353] Recchia F., Bernal J. D. K., Fong D. Y., Wong S. H. S., Chung P. K., Chan D. K. C., Capio C. M., Yu C. C. W., Wong S. W. S., Sit C. H. P., Chen Y.-J., Thompson W. R., Siu P. M. (2023). Physical activity interventions to alleviate depressive symptoms in children and adolescents a systematic review and meta-analysis. Jama Pediatrics.

[B8-behavsci-15-01353] Sina Education (2019). Development insights: Over 65% of parents believe sports should persist despite academic pressures.

[B29-behavsci-15-01353] Smith G., Williams L., O’Donnell C., McKechnie J. (2019). A series of n-of-1 studies examining the interrelationships between social cognitive theory constructs and physical activity behaviour within individuals. Psychology & Health.

[B30-behavsci-15-01353] Song Z. (2025). The influence mechanism of parental emotional support on adolescents’ emotion regulation strategies. 4th Academic Conference on Educational Information Technology Innovation and Development.

[B31-behavsci-15-01353] Sun H., Zhao X. (2011). Research report on the development of Chinese children and adolescents (1999–2010).

[B32-behavsci-15-01353] Tao J., Zhang X., Wang F., Gao Y., Cao X. (2024). The relationship between self-efficacy and physical activity among county school-aged children: The mediating role of exercise identity. Acta Universitatis Medicinalis Nanjing (Social Sciences).

[B33-behavsci-15-01353] Valcan D. S., Davis H., Pino-Pasternak D. (2018). Parental behaviours predicting early childhood executive functions: A meta-analysis. Educational Psychology Review.

[B34-behavsci-15-01353] Wan Y., Meng Y. (2024). Two decades of mental health research for chinese children and adolescents: Research hotspots, knowledge mapping, and development trends. Psychological Research.

[B35-behavsci-15-01353] Wang B. (2023). Companionship as education transcending anxiety: Evidence from research on family sports parenting styles. China Sport Science and Technology.

[B36-behavsci-15-01353] Wang M. (2019). An experimental study on the effectiveness of physical exercise in alleviating student anxiety. Huaxia Jiaoshi.

[B37-behavsci-15-01353] Wang Z. (1984). The SCL-90 symptom checklist. Shanghai Archives of Psychiatry.

[B38-behavsci-15-01353] Yang A., He C. (2024). The relationship between family physical education and mental health of students in compulsory education: The chain mediating role of parent-child communication and exercise behavior. Journal of Shenyang Sport University.

[B39-behavsci-15-01353] Zhu J., Mao Z. (2025). From physical education course to leisure time to promote physical activity of children and adolescents: Based on self-determination theory. Journal of the Capital Institute of Physical Education.

